# Bone in Human Penis

**Published:** 1913-11

**Authors:** 


BONE IN HUMAN PENIS. Arpad G. Gerster and F. S. Mandlebaum (sic) of New York, *Annals of Surgery,* June, 1913, report a case in a man aged 49, apparently due to pressure of straight front corset upon the dorsum. The specimen measured 3.5x1.7 c.m. and 2-3 m.m. in thickness. This is the sixteenth case in literature. Morpholgic relation to the normal bone of certain animals is denied, the formation being purely pathologic and due to mechanic irritation and various disease lesions. (Note. We have no sympathy with the patient, although, in the kind of a man who would wear a corset, the lesion may be considered conservative and not retributive.)

